# Ultrasound‐guided percutaneous metal‐organic frameworks based codelivery system of doxorubicin/acetazolamide for hepatocellular carcinoma therapy

**DOI:** 10.1002/ctm2.600

**Published:** 2021-10-14

**Authors:** Ziwei Jing, Xiaohui Wang, Na Li, Zhi Sun, Dingding Zhang, Lin Zhou, Fanxiang Yin, Qingquan Jia, Mengli Wang, Yaojuan Chu, Shuzhang Du, Yaping He, Qiuzheng Du, Xiaojian Zhang

**Affiliations:** ^1^ Department of Pharmacy The First Affiliated Hospital of Zhengzhou University Zhengzhou China; ^2^ School of Biological Science and Medical Engineering Southeast University Nanjing China; ^3^ Department of Ultrasound The First Affiliated Hospital of Zhengzhou University Zhengzhou China; ^4^ Department of Stomatology The First Affiliated Hospital of Zhengzhou University Zhengzhou China; ^5^ Department of Cardiovascular The First Affiliated Hospital of Zhengzhou University Zhengzhou China; ^6^ Translational Medicine Center The First Affiliated Hospital of Zhengzhou University Zhengzhou China

Dear Editor,

Currently, the enhanced permeability and retention (EPR) effect—one of the main mechanisms through which nanomedicines^1^ enter solid tumors after intravenous administration—has been mired in controversy due to its variation in different tumors^2^ and species^3^ (e.g., insufficient interendothelial gaps in human tumors compared with mouse models). In this study, a zeolitic imidazolate frameworks (ZIF‐8) nanocarrier, to facilitate codelivery of doxorubicin (DOX) and acetazolamide (ACE), was prepared using a one‐pot process and applied with ultrasound‐guided percutaneous intratumoral injection. Rational design of the (DOX+ACE)@ZIF‐8 considered the following facts: (a) after single‐dose percutaneous intratumoral injection under ultrasound guidance, the (DOX+ACE)@ZIF‐8 tends to directly accumulate at the tumor site and become internalized into tumor cells via endocytosis pathways; (b) ACE, a sulfonamide carbonic anhydrase (CA) inhibitor, currently used as antiepileptic, antiglaucoma, and diuretic agent,^4^, ^5^ may have a synergistic effect with DOX and its mechanism may be involved in the ability of inhibiting CA “to acidify the intratumoral environment”^6^, ^7^; and (c) with intratumoral administration route, the (DOX+ACE)@ZIF‐8 shows low systemic toxicity (Figure 1).

**FIGURE 1:**
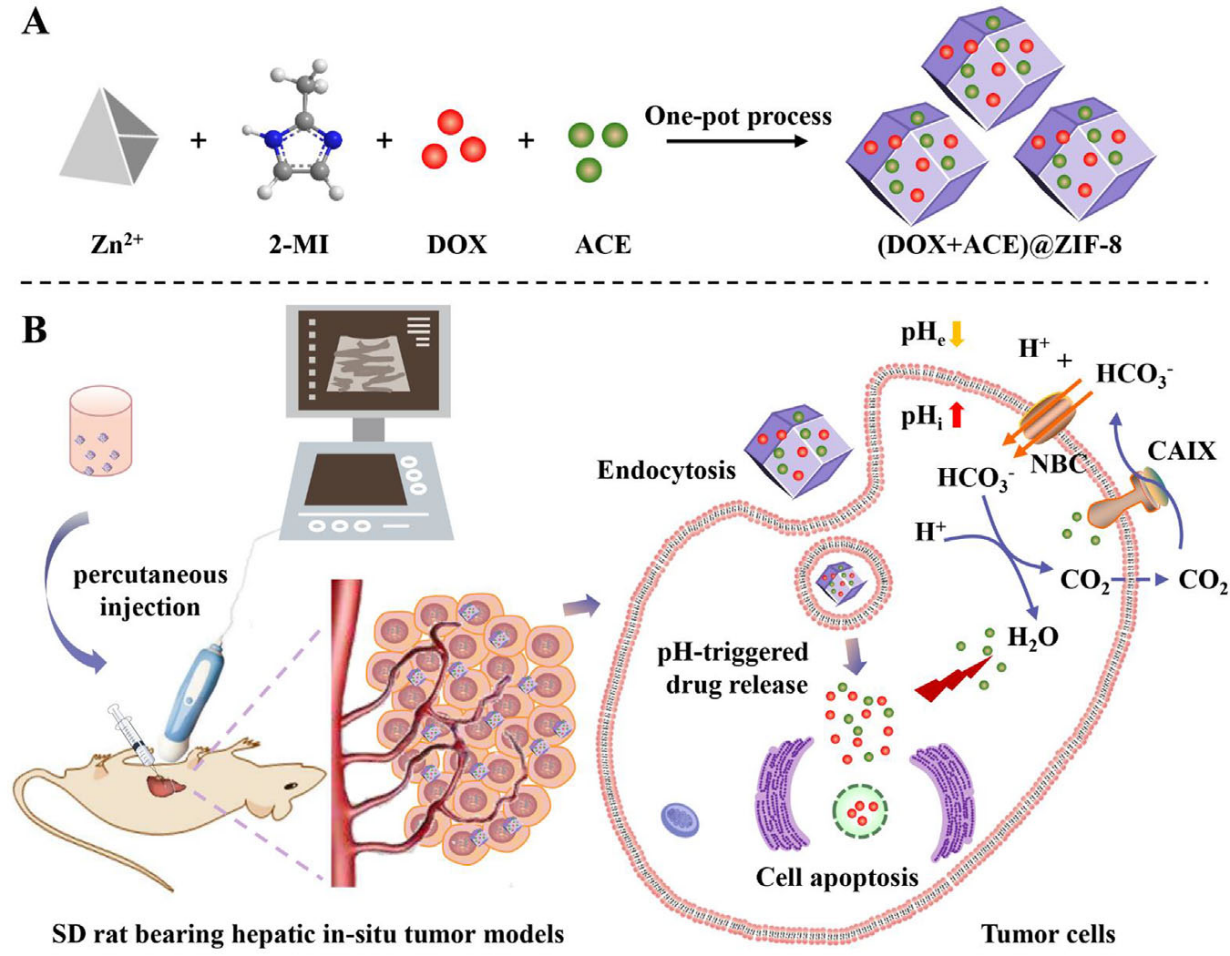
Construction and proposed mechanism of (DOX+ACE)@ZIF‐8 against the hepatic in‐situ tumor models. A, The preparation of metal‐organic frameworks nanocarrier‐based (DOX+ACE)@ZIF‐8 through one‐pot process. B, High tumor accumulation of (DOX+ACE)@ZIF‐8 through single‐dose percutaneous intratumoral injection under ultrasound guidance, followed by the cellular internalization via endocytosis pathways. At the tumor site, pH‐responsive released ACE inhibits CAIX from catalyzing CO_2_ to HCO_3_^−^ and H^+^ for “desensitizing” the cancer cells to the pH changes, thus enhancing the anticancer activity of DOX

With the advantages of the ZIF‐8 nanocarrier, our prepared (DOX+ACE)@ZIF‐8 has a particle size of 299.0 ± 18.7 nm and zeta potential of ‐2.46 ± 0.71 mV (Figure 2A; Figure S1). Similar to ZIF‐8, (DOX+ACE)@ZIF‐8 has a uniform cubic morphology (Figure 2B,C). Energy‐dispersive X‐ray spectroscopy (EDS) indicated that Zn/C values of (DOX+ACE)@ZIF‐8 were 16.45% (Figure 2D; Figures S2 and S3 and Table S1). Powder X‐ray diffraction (XRD) results and thermal gravimetric analysis (TGA) demonstrated that (DOX+ACE)@ZIF‐8 maintained crystal integrity^8^ and high thermostability (Figure 2E,F). Drug loading efficiency of (DOX+ACE)@ZIF‐8, calculated with dual‐wavelength spectrodensitometry, was 7.29% and 4.62% for DOX and ACE, respectively (Figure 2G, H; and Table S2). The pH‐responsive release behavior of (DOX+ACE)@ZIF‐8 was due to the protonated 2‐methylimidazole linkers, which destroyed the coordination of zinc ions and imidazolate rings (Figure 2I).^9^

**FIGURE 2:**
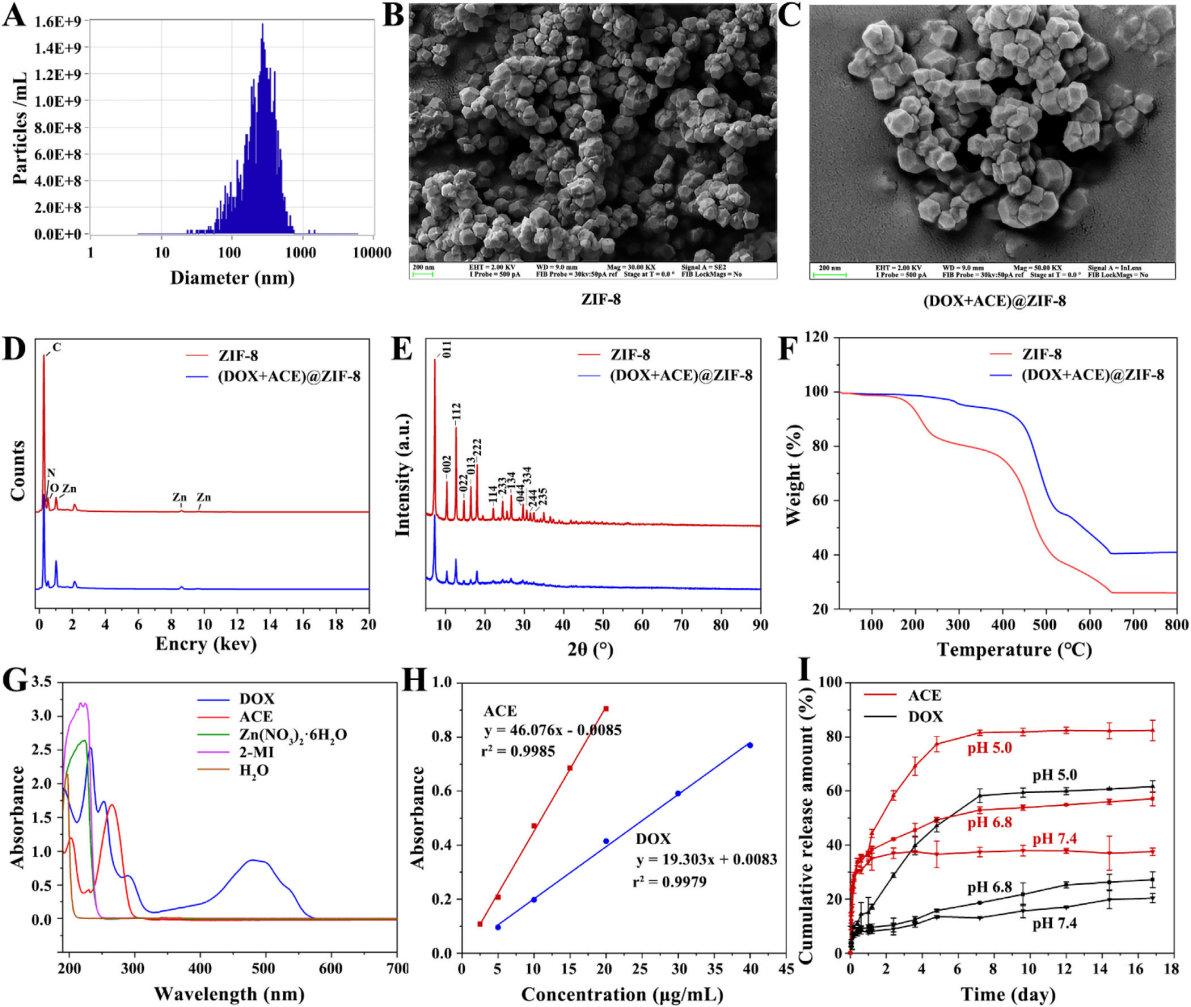
Characterization of (DOX+ACE)@ZIF‐8. A, Particle size distribution of (DOX+ACE)@ZIF‐8 measured by dynamic light scattering. B and C, Representative SEM images of ZIF‐8 and (DOX+ACE)@ZIF‐8; scale bars, 200 nm. D, Elemental distribution of ZIF‐8 and (DOX+ACE)@ZIF‐8 mapped by energy‐dispersive X‐ray spectroscopy. E, XRD spectra of ZIF‐8 and (DOX+ACE). F, TGA analysis of ZIF‐8 and (DOX+ACE). G, UV‐Vis DRS spectra of DOX, ACE, Zn(NO_3_)_2_, 2‐MI, and H_2_O. H, The standard curve of DOX at 480 nm and ACE at 262 nm. I, pH‐responsive release of DOX and ACE from (DOX+ACE)@ZIF‐8 in PBS (pH 5.0, 6.8, and 7.4) at 37°C

In vitro antitumor activity of (DOX+ACE)@ZIF‐8 against rat hepatoma Walker 256 cells was determined. First, the IC_50_ of blank ZIF‐8 (>100 μg/mL) to human normal liver cell line HL7702 showed low cytotoxicity of ZIF‐8 (Figure 3A). MTT results demonstrated that the antitumor efficacy of (DOX+ACE)@ZIF‐8 was dose‐dependent, and the IC_50_ values to Walker 256 cells were 2.36 μg/mL and 0.66 μg/mL (corresponding to ACE and DOX, respectively; Figure 3B). Moreover, wound healing and transwell assays showed that (DOX+ACE)@ZIF‐8 significantly inhibited Walker 256 cell motility (Figure 3C,E) and invasion (Figure 3D,F). The above results indicate that simultaneously loading ACE and DOX into a ZIF‐8 nanocarrier simultaneously is important for promoting the synergistic antitumor effect.^10^

**FIGURE 3:**
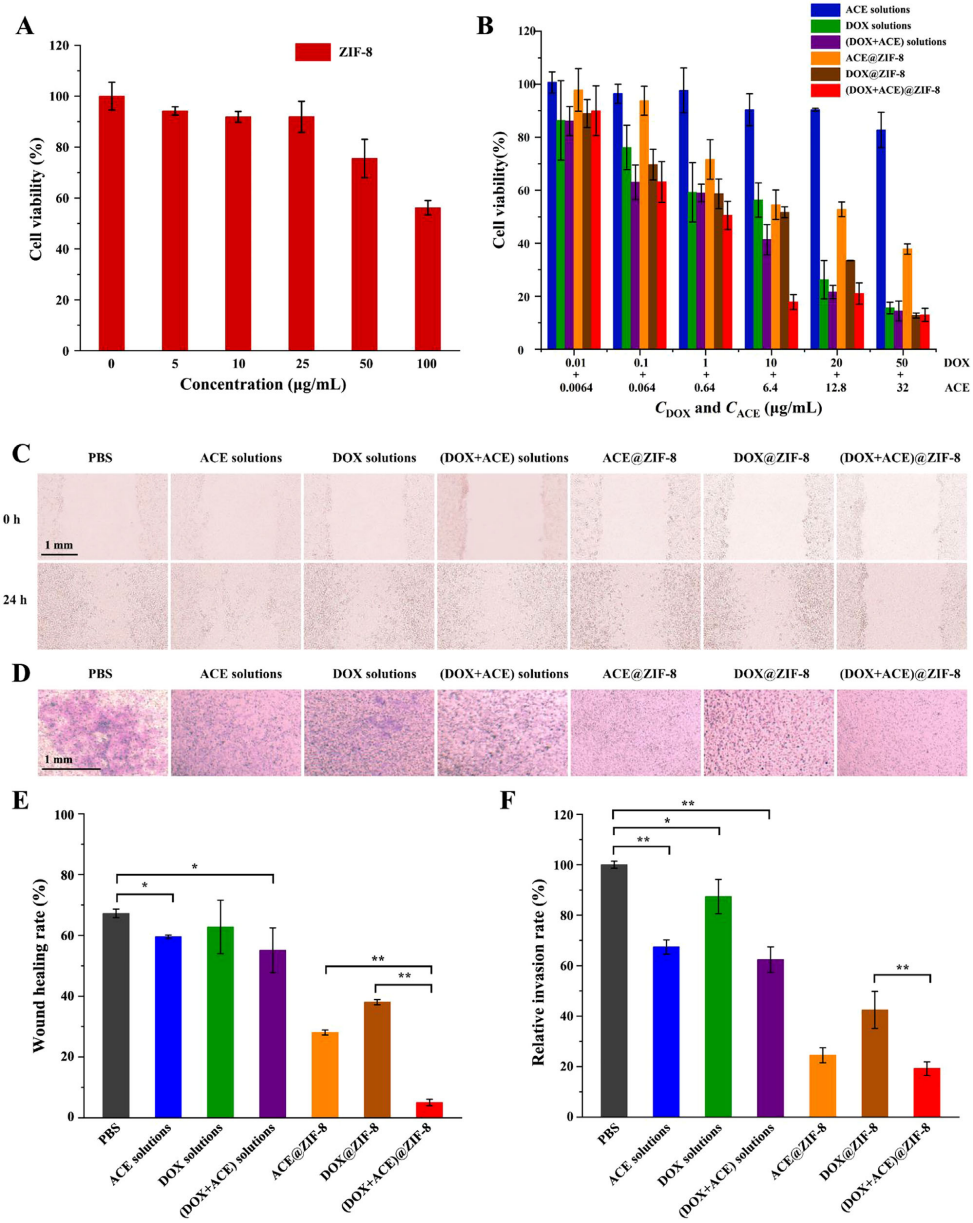
A, Cell inhibition rates of ZIF‐8 to HL7702 cells after 48 h of incubation (n = 5). B, Cell inhibition rates of ACE solutions, DOX solutions, (DOX+ACE) solutions, ACE@ZIF‐8, DOX@ZIF‐8, and (DOX+ACE)@ZIF‐8 to Walker 256 cells (n = 5). C and D, Representative images of cell migration and invasion of Walker 256 cells after treatment with PBS, ACE solutions, DOX solutions, (DOX+ACE) solutions, ACE@ZIF‐8, DOX@ZIF‐8, and (DOX+ACE)@ZIF‐8 for 24 h (n = 3). E and F, Wound healing rates and invasion rates of Walker 256 cells responding to wound healing assay (C) and transwell assay (D), respectively. Scale bar, 1 mm. *P < 0.05, **P < 0.01

Living cell imaging was applied to visualize the subcellular distribution of (DOX+ACE)@ZIF‐8 in real time. As the incubation time of (DOX+ACE)@ZIF‐8 with Walker 256 cells was prolonged, the yellow fluorescence intensity weakened, while the violet fluorescence was significantly enhanced, which showed that the released DOX from (DOX+ACE)@ZIF‐8 overlapped well with the cell nuclei stained by Hoechst 33258 (Figure S4 and Video Data S1). The endocytosis pathways of the (DOX+ACE)@ZIF‐8, combined with its pH‐sensitive drug release behavior, demonstrated that (DOX+ACE)@ZIF‐8 passed through the acidic lysosomes (pH 5–6), ensuring subsequent delivery of DOX into the cell nuclei while the cytoplasmic ACE recognized and interacted with CAIX simultaneously.

SD rats bearing hepatic in situ tumor models were established to verify the in vivo tumor inhibition capability of (DOX+ACE)@ZIF‐8. Figure 4A and B and Video Data S2 display the percutaneous injection procedure involving tumor model establishment. After drug administration, no significant differences in survival ratio were observed from the survival curves (Figure 4C). Bodyweight of each rat during the entire process was monitored (Figure 4D). Changes in the tumor volume measured by ultrasound are examined at each scheduled time (Figure 4E; Figure S5). Furthermore, a series of analyses were carried out including the dissected tumor volume, volume ratio, weight, and H&E staining of tumor‐bearing rats sacrificed at the end of the treatment (Figure 4F‐J; Figure S6). Notably, our prepared (DOX+ACE)@ZIF‐8, combining percutaneous intratumoral injection with ultrasound guidance, achieved significantly better tumor reduction than other groups with intratumoral or intravenous injection.

**FIGURE 4:**
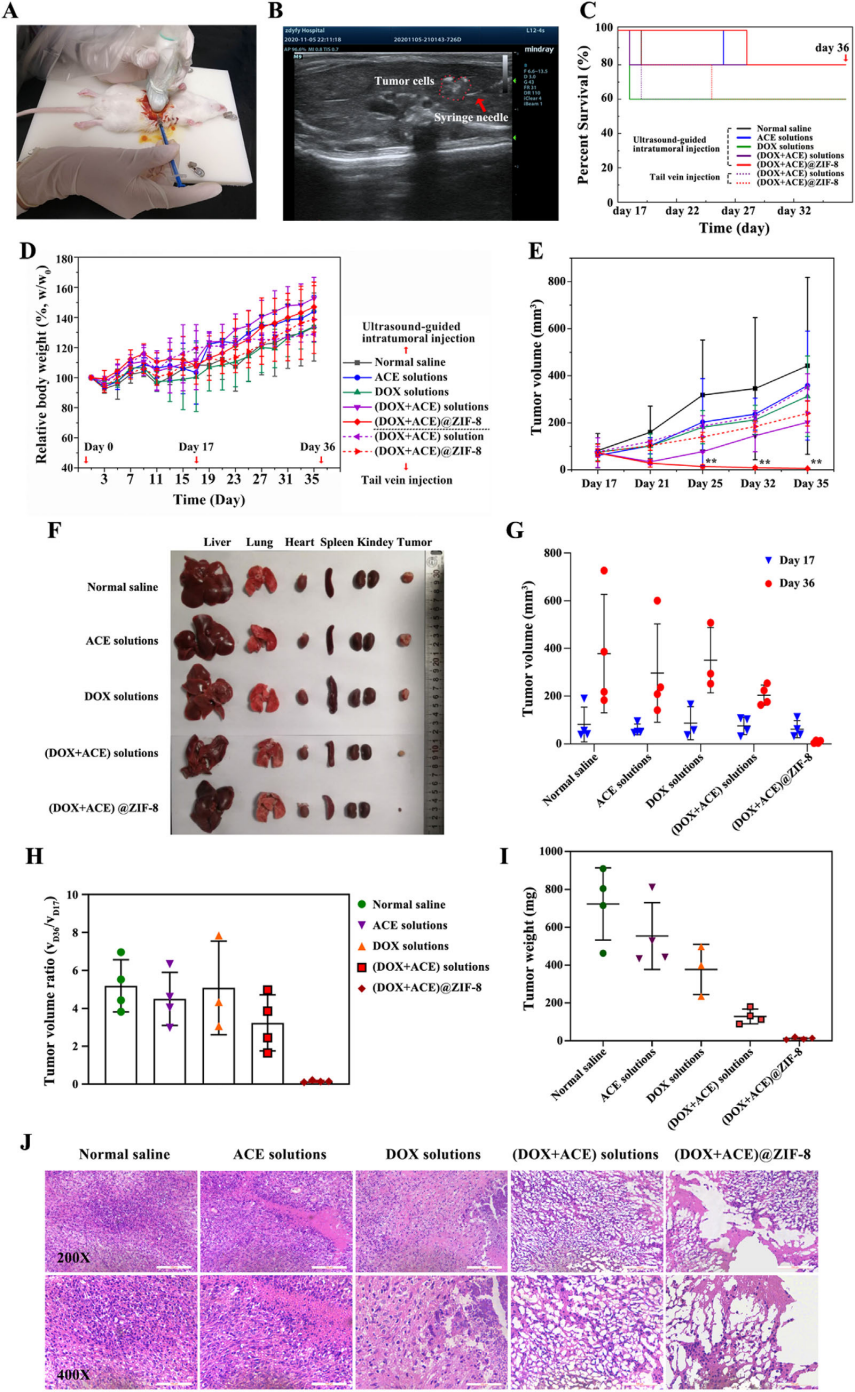
A, Photograph of the percutaneous injection procedure to establish the SD rats bearing hepatic in‐situ tumor models and drug administration under ultrasound guidance. B, Ultrasound imaging of percutaneous intratumoral injection of Walker 256 cells into the liver parenchyma of SD rats. The syringe needle (red arrow) and clusters of tumor cell suspensions (red dash line) in the liver parenchyma of SD rats were clearly observed by ultrasound imaging. C, Survival curves of the rats treated with different formulations. D, Changes in relative bodyweight of the rats during the in vivo experiment. E, Tumor volume of the rats as measured by ultrasound imaging. F, Photograph of tumors and major organs of the tumor‐bearing rats subjected to percutaneous intratumoral injection with different formulations. G, Tumor volume, H, tumor volume ratio, and I, tumor weight measured after SD rat anatomy. J, H&E staining of liver tumors after treatment, (n = 5 in per group). Day 1: animal model establishment; Day 17: treatment with single‐dose of formulations; Day 35: the last ultrasonic examination; Day 36: day of sacrifice. Scale bar, 100 μm. ^**^ *P* < 0.01

In vivo fluorescence imaging is a critical technology for the tracking of ZIF‐8 based nanocarriers. The drug distribution in isolated organs is shown in Figure S7. After intravenous injection, compared with IR783 solutions, the prolonged systemic circulation of IR783@ZIF‐8 was attributed to the EPR effect in rats, reflecting passive tumor‐targeted delivery. Interestingly, the rats subjected to percutaneous intratumoral injection of IR820@ZIF‐8 exhibited intensive fluorescent signals in the liver, indicating specific drug accumulation of (DOX+ACE)/ZIF‐8 at the tumor site.

In this respect, in vivo safety of (DOX+ACE)@ZIF‐8 was also evaluated. The (DOX+ACE)@ZIF‐8 with a concentration of 50–200 μg/mL exhibited negligible hemolytic potential (<5%) to red blood cells (Figure S8). After sacrifice, rats treated with single‐dosed percutaneous intratumoral or intravenous injection of (DOX+ACE)@ZIF‐8 showed almost no damage to normal tissue or adverse hematologic effects (Figure S9), preliminarily demonstrating the high biocompatibility of (DOX+ACE)@ZIF‐8.

In summary, a novel strategy by combining (DOX+ACE)@ZIF‐8 with ultrasound‐guided intratumoral injection, was proposed for HCC therapy. (DOX+ACE)@ZIF‐8 was shown to possess uniform cubic morphology, high thermal stability, and pH‐responsive release capability. After cellular internalization through the endocytosis pathway, (DOX+ACE)@ZIF‐8 specifically released the antitumor drug DOX and sulfonamide CA inhibitor ACE, thereby inhibiting the migration and invasion of Walker 256 cells as well as their cell viability in vitro, which ensured a synergistic antitumor effect. More importantly, compared with drug solutions or intravenous administration, (DOX+ACE)@ZIF‐8 exhibited superior antitumor efficacy in vivo with a single dose of percutaneous intratumoral injection under ultrasound guidance. In vivo real‐time fluorescence imaging indicated that (DOX+ACE)@ZIF‐8 increased drug accumulation at the tumor site. Finally, (DOX+ACE)@ZIF‐8 displayed good safety in vivo. This pioneering study proposes a new strategy for the development of nanomedicine with simple processing technology and a non‐intravenous administration route, which has potential for further clinical applications in the field of cancer treatment.

## CONFLICT OF INTEREST

The authors declare no conflict of interest.

## AUTHOR CONTRIBUTIONS

JZ and WX conceived the experiments; JZ, SZ, LN, ZD, ZL, and YF performed the in vitro experiments; JZ, WX, JQ, DS, WM, and CY performed the animal experiments; JZ, DQ, and HY analyzed the data; ZX conceptualized and designed the study, and supervised the project; all authors contributed to the manuscript and the discussion.

## Supporting information

Supporting information

Supporting information

Supporting information

## Data Availability

The data that support the findings of this study are available from the corresponding author upon reasonable request.
