# Supplementary material for: Ultrasound‐guided percutaneous metal‐organic frameworks based codelivery system of doxorubicin/acetazolamide for hepatocellular carcinoma therapy
Source: Clin Transl Med. 2021 Oct 14;11(10):e600. doi: 10.1002/ctm2.600 (PMC8516336; doi:10.1002/ctm2.600)
Supplement: Supplementary file 1 — Supporting information [file CTM2-11-e600-s001.docx]

**Supporting Information**

**Ultrasound-guided percutaneous metal organic frameworks-based codelivery system of doxorubicin/acetazolamide for hepatocellular carcinoma therapy**

Ziwei Jing^1,2#^, Xiaohui Wang^3#^, Na Li^4^, Zhi Sun^1^, Dingding Zhang^5^, Lin Zhou^1^, Fanxiang Yin^6^, Qingquan Jia^1^, Mengli Wang^1^, Yaojuan Chu^1^, Shuzhang Du^1^, Yaping He^1*^, Qiuzheng Du^1*^, Xiaojian Zhang^1*^

^1^ Department of Pharmacy, The First Affiliated Hospital of Zhengzhou University, Zhengzhou 450052, China.

^2^ School of Biological Science and Medical Engineering, Southeast University, Nanjing 210096, China

^3^ Department of Ultrasound, The First Affiliated Hospital of Zhengzhou University, Zhengzhou 450052, China

^4^ Department of Stomatology, The First Affiliated Hospital of Zhengzhou University, Zhengzhou 450052, China

^5^ Department of Cardiovascular, The First Affiliated Hospital of Zhengzhou University, Zhengzhou 450052, China

^6^ Translational Medicine Center, The First Affiliated Hospital of Zhengzhou University, Zhengzhou 450052, China

^#^ Ziwei Jing and Xiaohui Wang contributed equally to this study.

^*^ Corresponding authors: Prof Xiaojian Zhang, Zhengzhou University First Affiliated Hospital, No. 1, Jianshe East Road, Erqi District, Zhengzhou, Henan 450052, China. Email: zhangxiaojian_yxb@163.com

Dr. Qiuzheng Du, Zhengzhou University First Affiliated Hospital, No. 1, Jianshe East Road, Erqi District, Zhengzhou, Henan 450052, China. Email: duqiuzheng@163.com

Dr. Yaping He, Zhengzhou University First Affiliated Hospital, No. 1, Jianshe East Road, Erqi District, Zhengzhou, Henan 450052, China. Email: yapinghehyp@163.com

## Supplementary Materials

**1.1 Materials:** Zinc nitrate hexahydrate (Zn(NO_3_)_2_·6H_2_O, analytical grade) was purchased from Zhengzhou Paini Chemical Reagent Factory (Zhengzhou, Henan, China). 2-Methylimidazole was obtained from Shanghai Yien Chemical Technology Co., Ltd (Shanghai, China). Doxorubicin hydrochloride (DOX, 98%) and acetazolamide (ACE, 99%) were obtained from Macklin Co., Ltd (Shanghai, China). Dulbecco’s modified Eagle medium (DMEM), fetal bovine serum (FBS), trypsin-EDTA, penicillin and streptomycin were purchased from Gibco (Grand Island, NY, USA). Dialysis bag (Mw = 1 kDa, 60 mm) and 3-(4, 5,-dimethylthiazol-2-yl)-2, 5-diphenyltetrazolium bromide (MTT) were purchased from Solarbio Technology Co., Ltd (Beijing, China). BD Matrigel was obtained from BD Biosciences (San Jose, CA, USA). 4% Paraformaldehyde was purchased from Wuhan Seville Biotechnology Co., Ltd (Wuhan, Hubei, China). Hoechst 33258 was purchased from US Everbright® Inc. (Suzhou, Jiangsu, China). LysoTracker Green was obtained from Shanghai Beyotime Biotechnology Co., Ltd (Shanghai, China). IR783 was bought from Aladdin Biochemical Technology Co., Ltd. (Shanghai, China). All chemicals and reagents were commercially available and used without further purifcation.

**1.2 Cell and animal:** Rat hepatoma Walker 256 cells and human normal liver HL7702 cells were purchased from American Type Culture Collection. Male SD rats (6 weeks of age, 180 ± 20 g) were obtained from the laboratory animal center of Zhengzhou University (Zhengzhou, Henan, China). All animals were housed with free access to water and feed, and maintained in the standard temperature and light controlled animal facility. The operations were undertaken comply with the National Institute of Health Guide for the Care and Use of Laboratory Animals, with the approval of the Medical Ethics Committee of the First Affiliated Hospital of Zhengzhou University, Zhengzhou, China (2020-KS-HNSR289).

**1.3 Synthesis of (DOX+ACE)@ZIF-8:** (DOX+ACE)@ZIF-8 was synthesized according to previous study. ^1^ Typically, 16 mg of ACE was heated and dissolved in 8 mL of boiling deionized water, 16 mg of DOX and 2 g of 2-methylimidazole were added under vigorous stirring. Then 0.8 mL of Zn(NO_3_)_2_ solution (0.2g) was drop-wise added into the mixed solution. After being stirred for 30 min, the obtained suspensions were centrifugated (10000 rpm, 10 min) and disposed the supernatant, the generated (DOX+ACE)@ZIF-8 were then washed with water/methanol (1:1) and finally dried by lyophilization. Moreover, ZIF-8, DOX@ZIF-8, ACE@ZIF-8 and IR783@ZIF-8 were synthesized for comparison with 8 mL H_2_O, DOX solutions, ACE solutions or IR783 solutions.

**1.4 Characterizations of (DOX+ACE)@ZIF-8:** The particle size and zeta potential were determined by the dynamic light scattering method (Fullerton, California, USA) at 25°C. The appearance and interface of (DOX+ACE)@ZIF-8 were characterized by electron microscopy from Japan, including scanning electron microscopy (SEM, S-3400NII) with energy-disperse X-ray spectroscopy (EDS). The crystal structures of the specimens were analyzed by the powder X-ray diffraction (XRD, Bruker D8, Germany) patterns with Cu Kα radiation, operating at a 2θ range from 5° to 90° with the fast scanning mode at wide-angle. The thermal gravimetric analysis (TGA) was performed with a heating rate of 10°C/min from 25°C to 800°C. The drug loading efficiency of (DOX+ACE)@ZIF-8 was evaluated by the UV-Vis spectrophotometer (UV-1800, Shanghai Macy Instrument Co., Ltd, China).

**1.5 Release of drugs from (DOX+ACE)@ZIF-8:** The in vitro release behavior of (DOX+ACE)@ZIF-8 was study with a typical release system. The (DOX+ACE)@ZIF-8 (5 mL, at a dose of DOX of 700 µg and ACE of 444 µg) was dispersed into dialysis bags, which was immersed in PBS (pH 5.0, 6.8 and 7.4, 100 mL, respectively) and incubated in a shaking bed at 37°C. The release media (4 mL) were collected at each time point, while the same volume of medium was replenished. The amount of released DOX and ACE was determined by UV-Vis spectrophotometry. This drug release experiment was performed in triplicate.

**1.6 In vitro** **cytotoxicity assay:** The in vitro antitumor effect of (DOX+ACE)@ZIF-8 was evaluated in Walker 256 cells using the MTT assays. Walker 256 cells were grown in DMEM supplemented with 10% FBS. The cells were seeded onto 96-well plates with 1 × 10^4^ cells per well to preculture overnight. A fresh medium that contained the samples (PBS, ACE solutions, DOX solutions, (DOX+ACE) solutions, ACE@ZIF-8, DOX@ZIF-8 or (DOX+ACE)@ZIF-8) at various concentrations was added. After additional incubation of 48 h, 10 μL of 5 mg/mL MTT was then added. After 4 h, the supernatant was replaced by 150 µL of DMSO. The absorbance (A) was measured by a Bio-Rad Microplate Reader (SpectraMax 190, Molecular Devices Co., Ltd) at 490 nm. The cell inhibition rates were calculated using the following Equation (1).

| Cell viability (%) = | A _sample_-A _blank_ | × 100% | (1) |
| --- | --- | --- | --- |
|  | A _control_-A _blank_ |  |  |

**1.7 In vitro** **cell migration and invasion assays:** For the wound healing assay, 1 × 10^6^ Walker 256 cells were seeded onto cell culture dish (60 mm, Corning, USA). After incubation for 24 h, a scratch wound was made with a sterile pipette tip (200 µL) and the cells were washed with PBS. Afterwards, the cells were incubated with PBS, ACE solutions, DOX solutions, (DOX+ACE) solutions, DOX@ZIF-8, ACE@ZIF-8 or (DOX+ACE)@ZIF-8 (at an equivalent DOX concentration of 1 µg/mL and ACE concentration of 0.64 µg/mL) for 24 h. An inverted optical microscope (Leica DMi1, Wetzlar, Germany) was employed to obtain the imagine at 0 and 24 h, and the gaps distance was measured. The wound healing rate was calculated according to Equation (2).

| Wound healing rate (%) = | Distance at t_i_ | × 100% | (2) |
| --- | --- | --- | --- |
|  | Distance at t_0_ |  |  |

For the transwell assay, 1 × 10^5^ Walker 256 cells were seeded onto the top chamber of a transwell insert (24 well insert, pore size, 8 µm, Corning, USA), which was precoated with 60 µL of Matrigel. Subseqently, 100 µL of serum free medium diluted with PBS, ACE solutions, DOX solutions, (DOX+ACE) solutions, DOX@ZIF-8, ACE@ZIF-8 or (DOX+ACE)@ZIF-8 (at an DOX concentration of 1 µg/mL and ACE concentration of 0.64 µg/mL) was added to the upper chamber, and the lower chambers were filled with 600 µL of 20% serum medium. After incubation for 48 h, the remaining cells in the upper chambers were removed, while the cells that migrated to the lower chamber were fixed in 4% paraformaldehyde for 15 min and stained with crystal violet. Then, an inverted microscope was used to obtain the images of the cells in each lower chamber. Finally, the cells stained with crystal violet were eluted with 33% acetic acid and measured at 570 nm using a Microplate Reader. The relative invasion rate was calculated using Equation (3).

| Relative invasion rate (%) = | A _sample_ | × 100% | (3) |
| --- | --- | --- | --- |
|  | A _PBS control_ |  |  |

**1.8 Colocalization of cellular (DOX+ACE)@ZIF-8:** 2 × 10^5^ Walker 256 cells in 10% serum medium were seeded in the glass bottom cell culture dish (20 mm, Wuxi Nest Biotechnology Co., Ltd). After incubation for 24 h, the cells were treated with LysoTracker Green for 20 min and washed with PBS three times for lysosome staining. Subsequently, the cells were treated with Hoechst 33258 for 15 min and washed with PBS three times for nucleus staining. Then, 2 mL of fresh medium that contained (DOX+ACE)@ZIF-8 (at an equivalent DOX concentration of 5 µg/mL and ACE concentration of 3.2 µg/mL) was added to the cell culture dish. Cell internalization and the intracellular colocalization of (DOX+ACE)@ZIF-8 in Walker 256 cells was observed using GE DeltaVision OMX SR (Applied Precision Co., Ltd, USA).

**1.9 Ultrasound guided percutaneous injection of Walker 256 cells into liver parenchyma of SD rat:** The hepatic in-situ tumor models had been established by percutaneous injecting Walker 256 cells into the liver parenchyma of SD rats under ultrasound guidance. Briefly, the SD rats were anesthetized with 10% chloral hydrate. The abdomen was shaved and a preliminary ultrasound (Mindray M9cv, Midray Medical Intl Ltd, China) was performed with a L12-4S transducer for visualization and determination of the target implantation site within the liver. Next, Walker 256 cells were dispersed in normal saline at 2.0 × 10^6^ cells/mL. And then 0.1 mL of cells suspensions was slowly injected into the liver with 1 mL syringe under direct ultrasound guidance. Finally, the ultrasound probe was used to compress the abdominal wall of the needle hole side for about 1 min to reduce bleeding and cell suspension leakage. The SD rats were evaluated daily for weight, mortality and tumor volume (Equation 4).

| Tumor volume = | *L* ×*W* ^2^ |  | (4) |
| --- | --- | --- | --- |
|  | 2 |  |  |

**1.10** **In vivo antitumor efficacy of (DOX+ACE)@ZIF-8:** The in vivo antitumor efficacy of different formulations (normal saline, ACE solutions, DOX solutions, (DOX+ACE) solutions and (DOX+ACE)@ZIF-8), 5 animals in per group) and different administration routes were evaluated on the hepatic in-situ tumor models. Treatments were started when the tumor volumes of rats reached 50-100 mm^3^ (at day 17). For percutaneous intratumoral injection under ultrasound guidance, each group was treated by 50 µL of normal saline, ACE solutions, DOX solutions, (DOX+ACE) solutions and (DOX+ACE)@ZIF-8 (at DOX concentration of 100 µg/mL and ACE concentration of 64 µg/mL), respectively. For intravenous injection, the other two groups were tail vein injected with 50 µL of (DOX+ACE) solutions or (DOX+ACE)@ZIF-8, respectively. The weight and mortality of rats of the seven groups were monitored daily, the tumor volume was measured by ultrasonic examination at each scheduled time.

At day 36, all of the SD rats were sacrificed. The tumor and major organs (liver, heart, spleen, kidney and lung) were obtained to examine the in vivo antitumor efficacy and security of the different formulations. These isolated organs were washed with normal saline and soaked in the 4% formaldehyde. For the histopathological analysis, hematoxylin and eosin (H&E) were used to stain the paraffin embedded tissue.

**1.11 In vivo** **fluorescence imaging and drug biodistribution:** The hydrophilic dye IR783 was used to prepare IR783@ZIF-8 through the method similar to (DOX+ACE)@ZIF-8. The tumor-bearing SD rats was used to study the drug distribution of IR783@ZIF-8 (1.5 mg/kg). Briefly, the rats received 50 µL of IR783 solutions or IR783@ZIF-8 by percutaneous intratumoral injection under ultrasound guidance or intravenous injection.

The rats were sacrificed after 24 h. The liver, heart, spleen, kidney and lung were carefully dissected, and their fluorescence images were collected by Xenogen IVIS Kinetic system, and then analyzed by IVIS Living Image 3.1 software.

**1.12 Safety evaluation of (DOX+ACE)@ZIF-8:** In vitro cytotoxicity of ZIF-8 (0, 5, 10, 25, 50 and 100 μg/mL) was carried out by the MTT assay with human normal liver HL7702 cells. The in vivo safety of (DOX+ACE)@ZIF-8 was initially investigated with hemolysis test, H&E histology staining (as mentioned above) and serum biochemical analysis.

Before in vivo study, the red blood cells (RBCs) hemolysis in presence of (DOX+ACE)@ZIF-8 was examined. Freshly collected blood from SD rats was centrifuged to remove the serum. The obtained RBCs were washed with normal saline and then dispersed to prepare the 2% (v/v) RBCs suspension. Then, (DOX+ACE)@ZIF-8 with different concentrations (50, 100, 150 and 200 µg/mL, 1.5 mL) was mixed with 1.5 mL of 2% RBCs suspension, 0.9% NaCl solution and H_2_O were regarded as negative and positive controls, respectively. The mixtures were incubated for 3 h and centrifuged for 15 min. The supernatant absorbance was measured with UV-Vis spectrophotometer at 541 nm. ^1,2^ The hemolysis ratios of (DOX+ACE)@ZIF-8 were calculated using Equation (5).

| Hemolysis ratio (%) = | A _sample_ - A _negative control_ | × 100% | (5) |
| --- | --- | --- | --- |
|  | A _positive control_ - A _negative control_ |  |  |

At the end of the treatment, serum of the tumor-bearing rat was collected to determine the ALT and AST levels (Chemray 800, Rayto, China).

**1.13 Statistical analysis:** All data were expressed as “means and standard deviations”, indicated as “means ± SD”. One-way ANOVA of statistical analysis systems was applied for the statistical analysis in different groups. The levels of statistical differences were represented when the “p” values were <0.05.

## Supplementary References

1. Wu, M. X.; Yang, Y. W., Metal-Organic Framework (MOF)-Based Drug/Cargo Delivery and Cancer Therapy. Adv Mater. 2017, 29, 1606134.

2. He, Y.; Xiong, T.; He, S.; Sun, H.; Huang, C.; Ren, X.; Wu, L.; Patterson, L. H.; Zhang, J., Pulmonary Targeting Crosslinked Cyclodextrin Metal–Organic Frameworks for Lung Cancer Therapy. Adv Funct Mater. 2021, 31, 2004550.

## Supplementary Figures


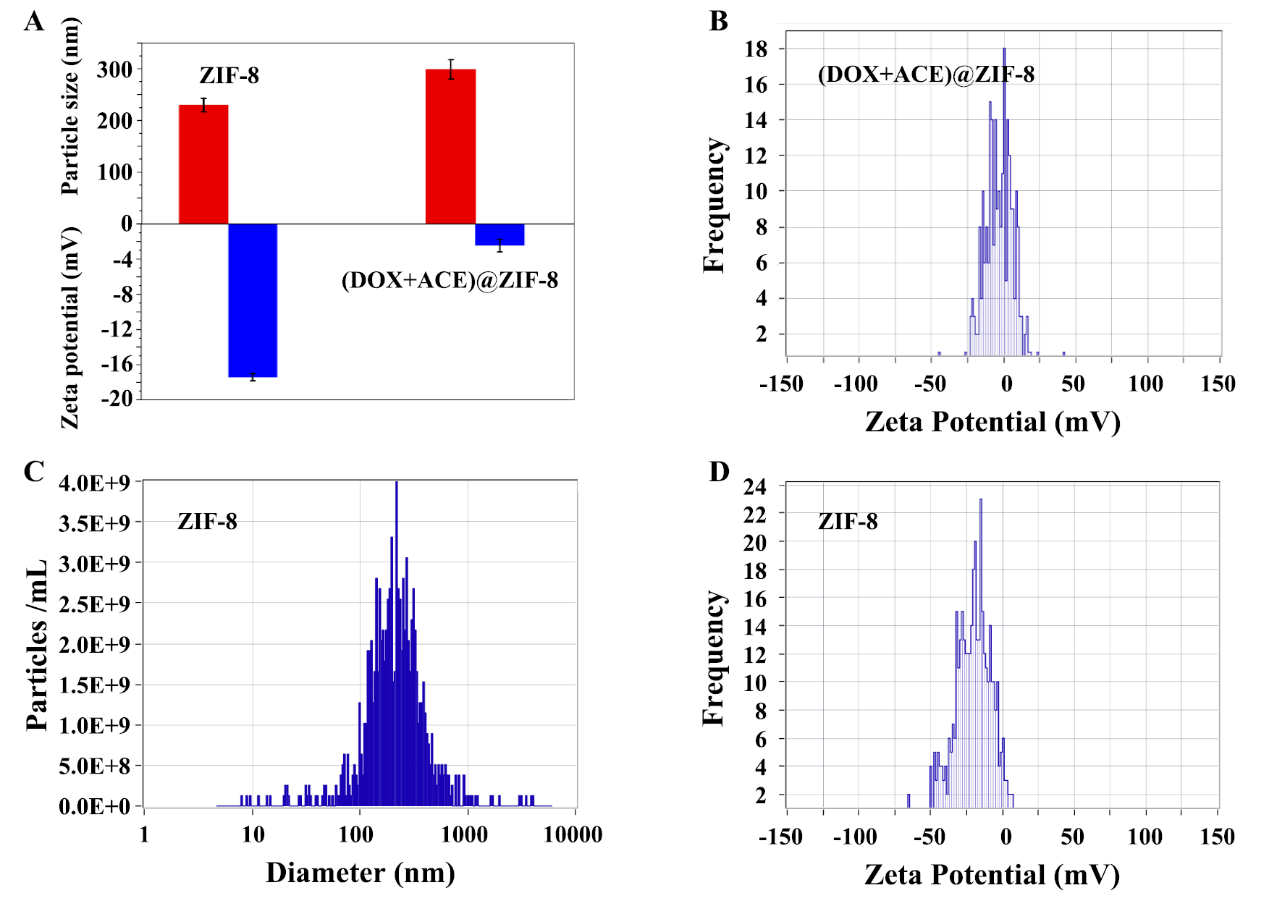


**Figure S1.** Particle size and zeta potential of (DOX+ACE)@ZIF-8 (A, B) and ZIF-8 (A ,C, D) measured by dynamic light scattering.


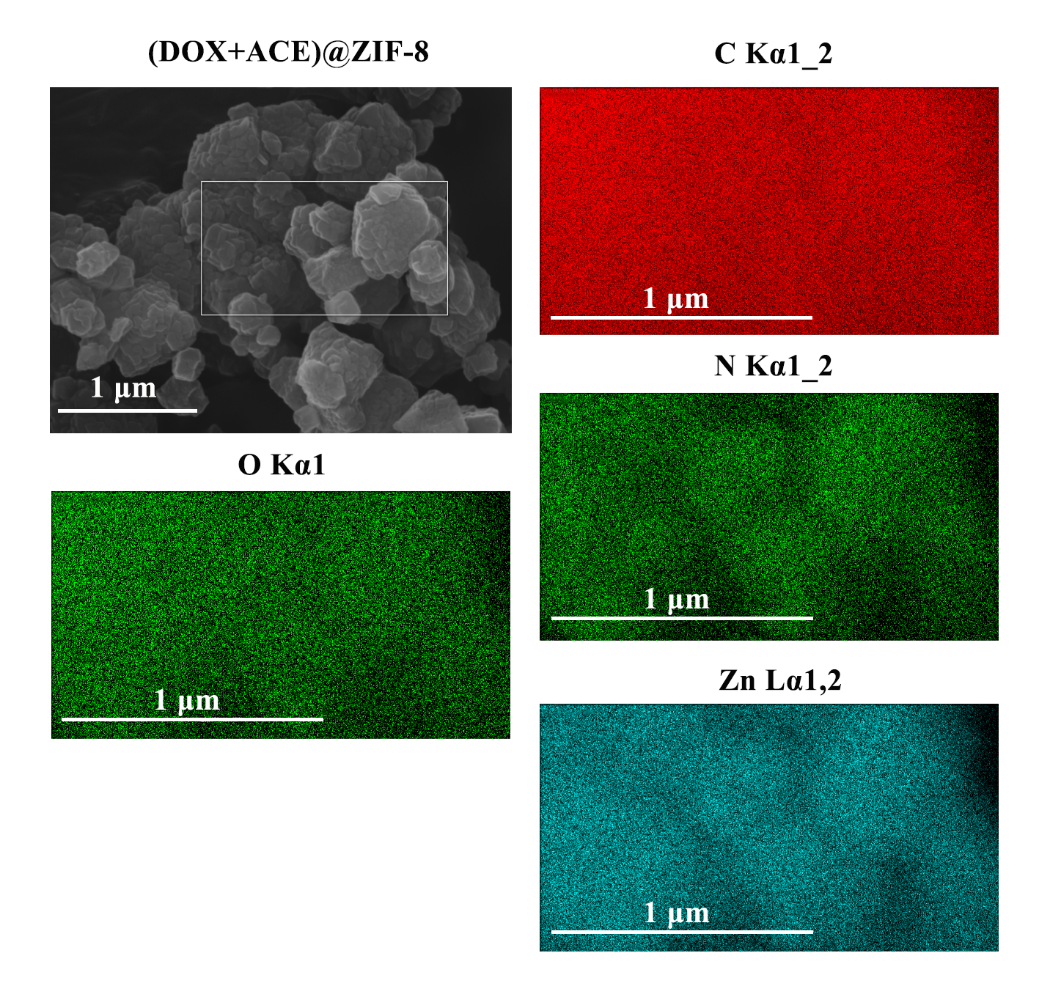


**Figure S2.** Elemental mapping image of C, N, O and Zn element image of (DOX+ACE)@ZIF-8.


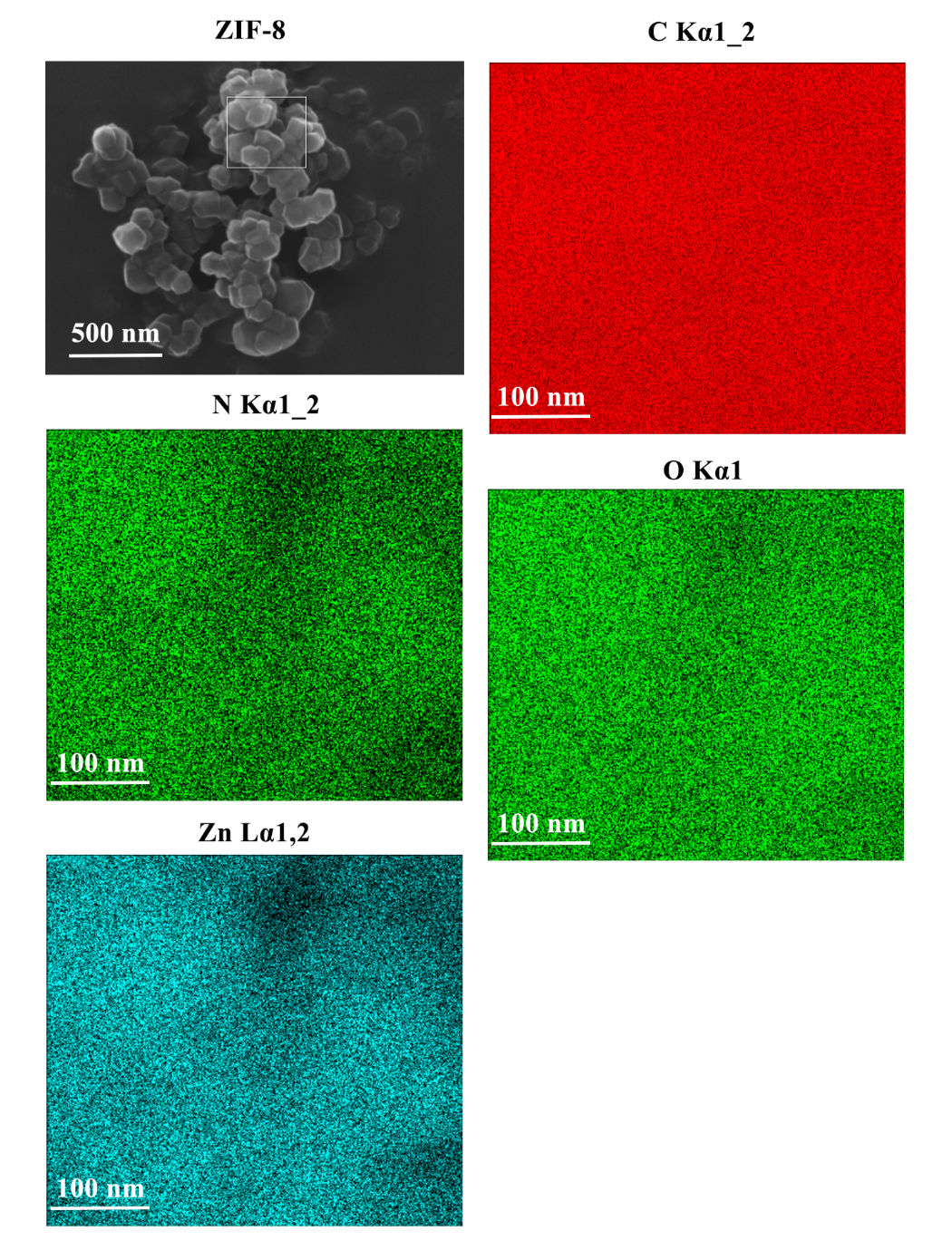


**Figure S3.** Elemental mapping image of C, N, O and Zn element image of ZIF-8.


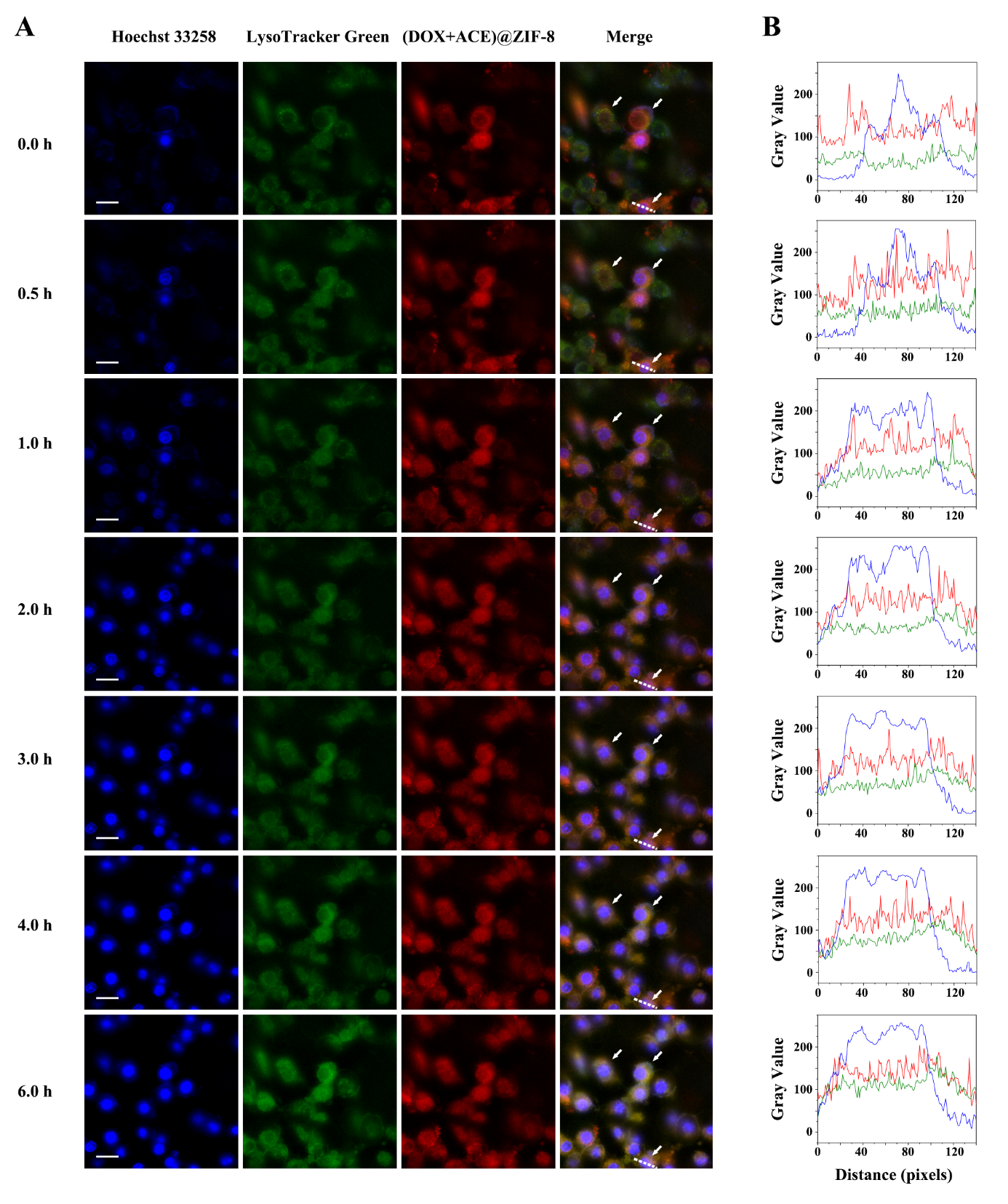


**Figure S4.** A) The representative fluorescence images showing co-localization of (DOX+ACE)@ZIF-8 with lysosomes and cell nucleus of Walker 256 cells by DeltaVision Ultra. B) Intensity curves measured along a vector across three fluorescent channel, white short dash as shown in (A). Hoechst 33258 = blue channel (cell nucleus was stained with Hoechst 33258), LysoTracker Green = green channel (lysosome was stained with LysoTracker Green), (DOX+ACE)@ZIF-8 = red channel. Merge = the overlapping images of blue, green and red channels. Scale bars = 15 μm.


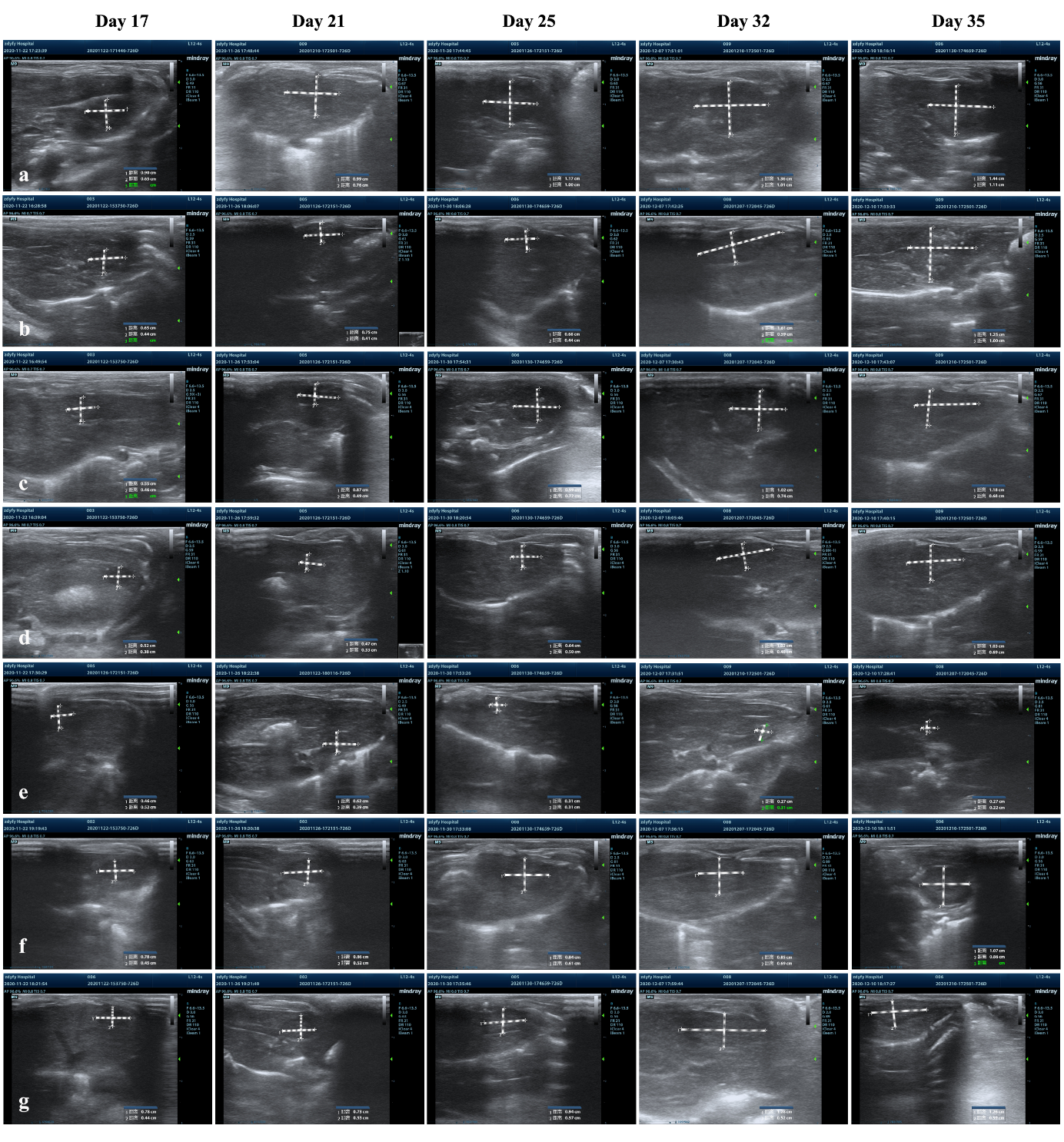


**Figure S5.** The representative ultrasound imaging of rat after treatment with different formulations at each scheduled time (n=5). a-e: group of normal saline, ACE solutions, DOX solutions, (DOX+ACE) solutions and (DOX+ACE)@ZIF-8, respectively, administrated by intratumor injection; f-g: group of (DOX+ACE) solutions and (DOX+ACE)@ZIF-8, administrated by intravenous injection.

**
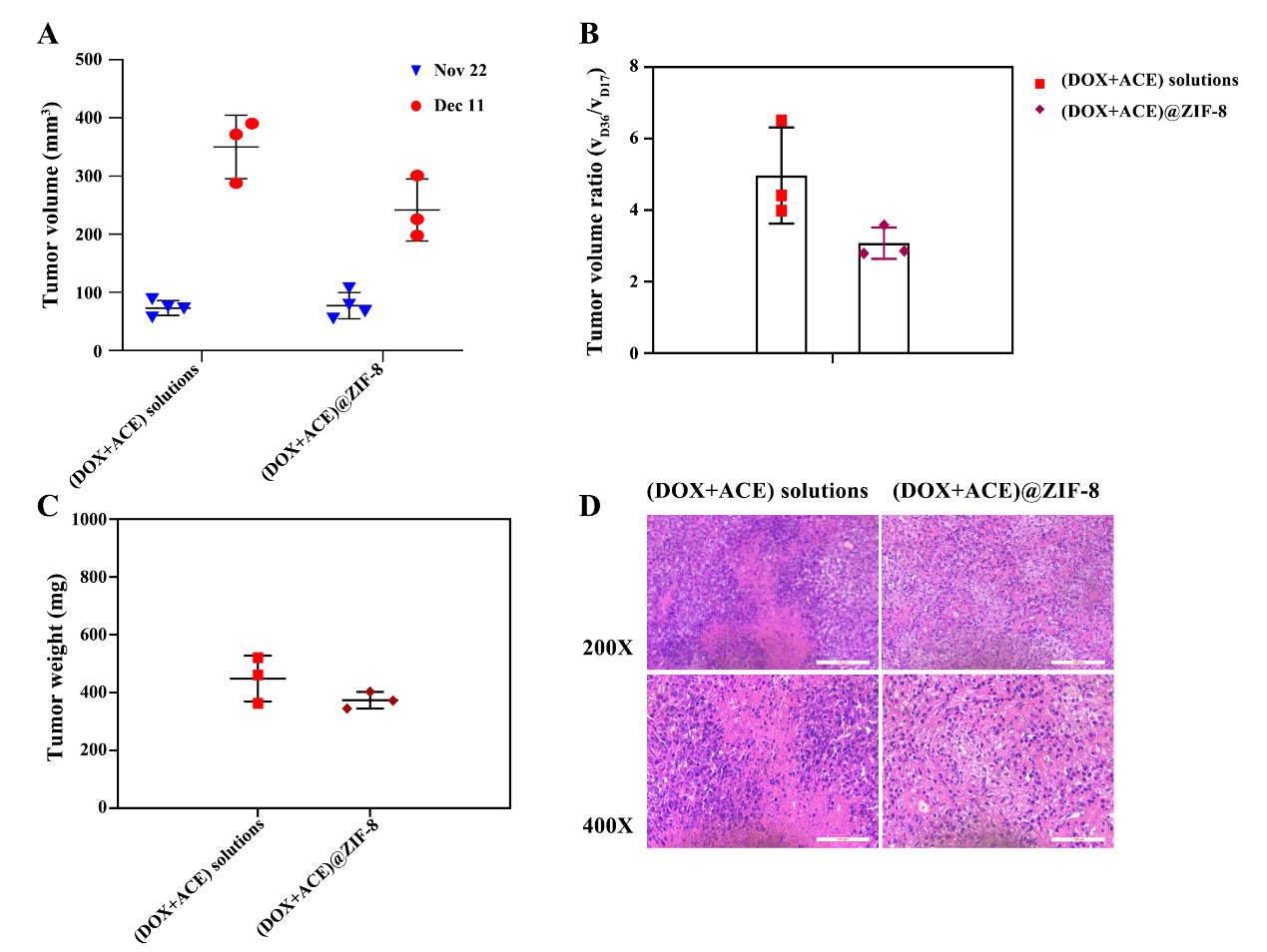
**

**Figure S6.** A) Tumor volume, B) tumor volume ratio and C) tumor weight of the tumor-bearing rats intravenous injected of (DOX+ACE) solutions or (DOX+ACE)@ZIF-8. Nov 22, represents before the treatment; Dec 11, represents after the treatment. E) H&E histology staining of liver tumors after treatment. Scale bar, 100 µm.


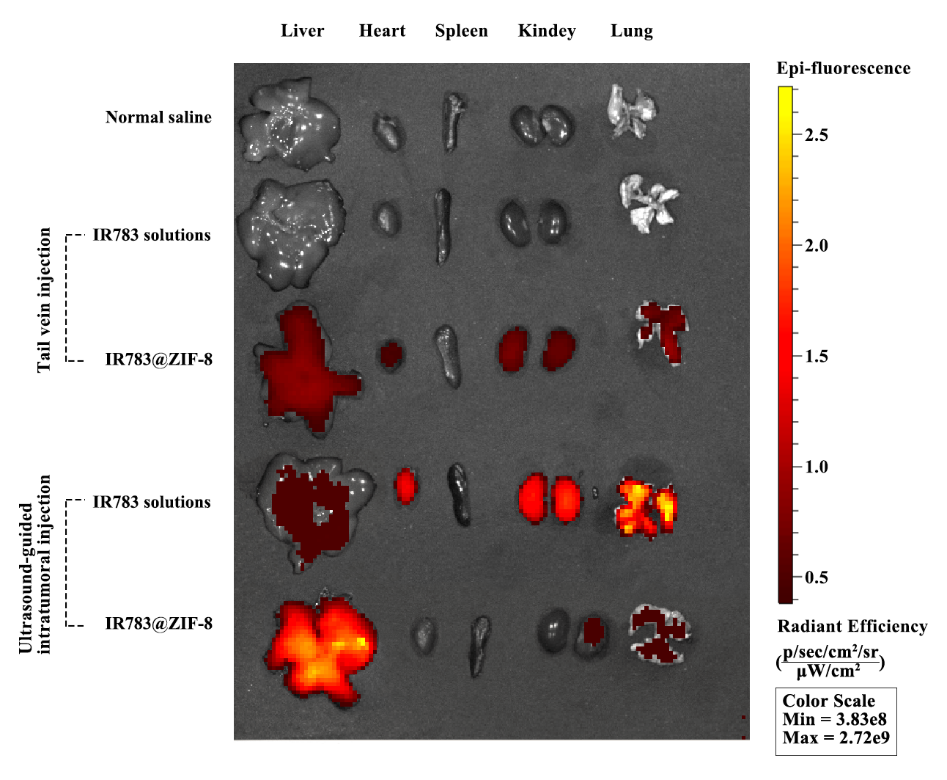


**Figure S7**. Distribution of IR783 and IR783@ZIF-8 in major organs of tumor-bearing SD rats after percutaneous intratumoral or intravenous injection at 24 h.


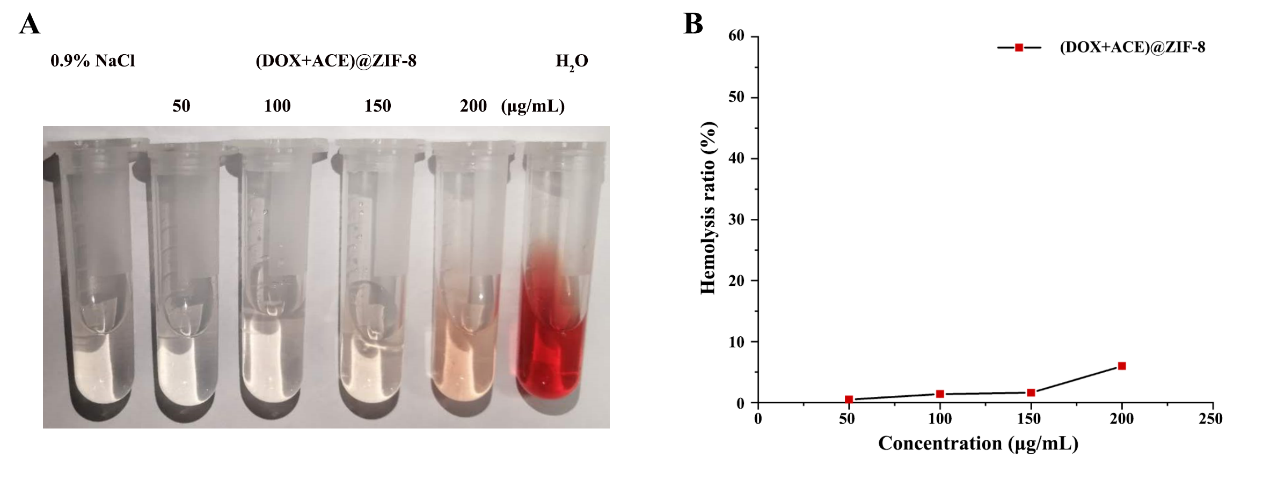


**Figure S8.** A) The photo (A) and Hemolysis ratio (%) of Red blood cells after treatment with 0.9 NaCl or (DOX+ACE)@ZIF-8 (50, 100, 150 and 200 μg/mL). (n=3).


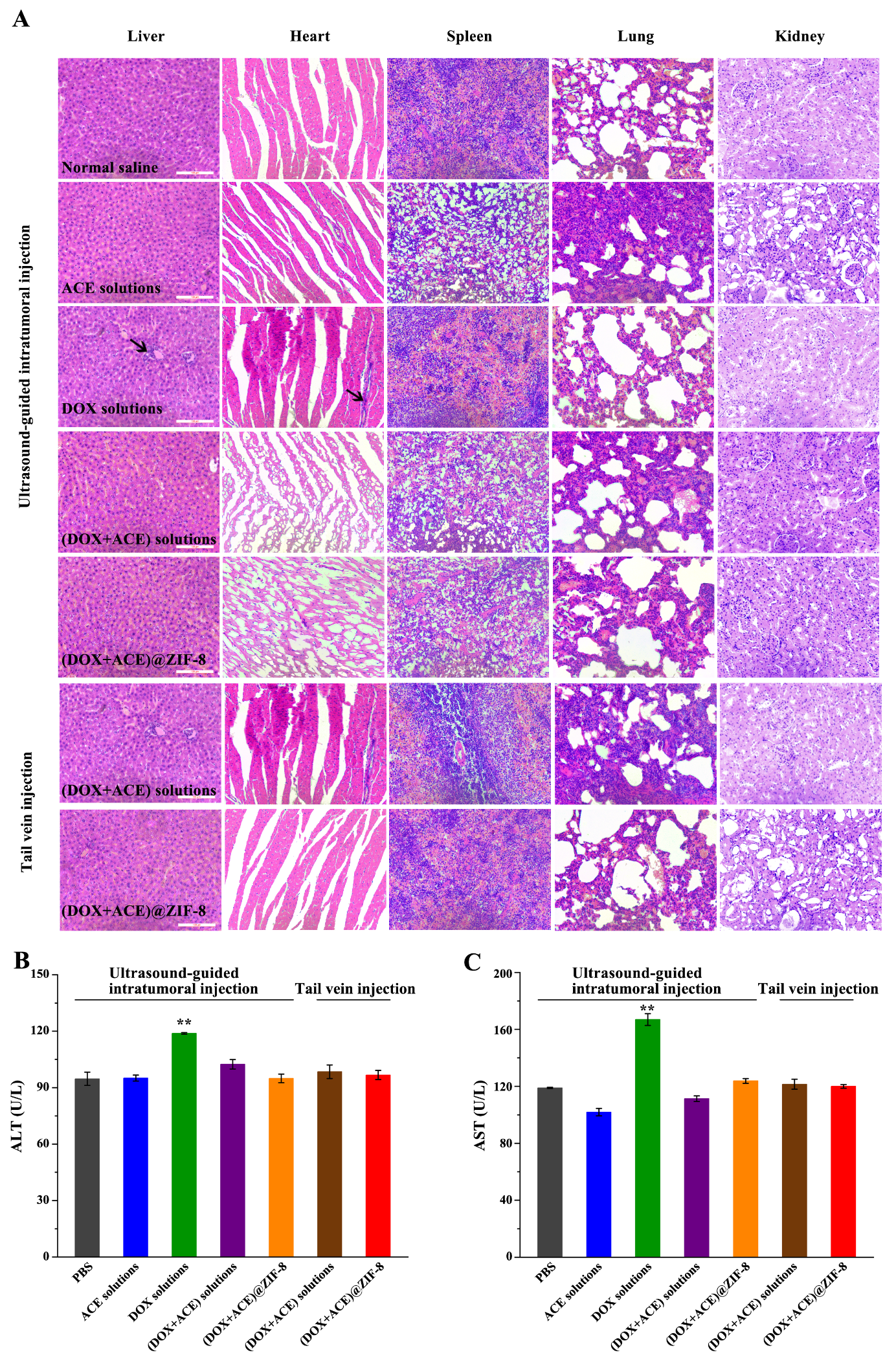


**Figure S9**. A) H&E histology of major organs collected from SD rats bearing hepatic in-situ tumor models after the treatment with different formulations by intravenous injection. Scale bar, 100 µm. B) Determination of serum alanine aminotransferase (ALT) and aspartate aminotransferase (AST) levels after the treatment. **p < 0.01.

**Table S1.** The chemical composition for (DOX+ACE)@ZIF-8 and ZIF-8 from EDS.

| Sample | Elements | | | | Atomic percentage (%)  (Zn/C) |
| --- | --- | --- | --- | --- | --- |
|  | C | N | O | Zn |  |
| (DOX+ACE)@ZIF-8 | 79.74 | 0.00 | 7.14 | 13.12 | 16.45% |
| ZIF-8 | 83.32 | 0.00 | 11.30 | 5.37 | 6.45% |

**Table S2.** The drug loading content for DOX@ZIF-8, ACE@ZIF-8 and (DOX+ACE)@ZIF-8 measured by UV-VIS spectrophotometry (n=3).

| Sample | Drug loading efficiency | |
| --- | --- | --- |
|  | DOX (%) | ACE (%) |
| DOX@ZIF-8 | 10.24 ± 0.01% | - |
| ACE@ZIF-8 | - | 4.85 ± 0.01% |
| (DOX+ACE)@ZIF-8 | 7.29 ± 0.04 % | 4.62 ± 0.01 % |

**Table S3.** The IC50 values of ACE solutions, DOX solutions, (DOX+ACE) solutions, ACE@ZIF-8, DOX@ZIF-8 and (DOX+ACE)@ZIF-8 incubated with walker 256 cells for 48 h (n=5).

| Sample | IC50 values (μg/mL)  (ACE) | IC50 values (μg/mL)  (DOX) |
| --- | --- | --- |
| ACE solutions | 4748.59 | - |
| DOX solutions | - | 2.63 |
| (DOX+ACE) solutions |  | 1.17 |
| ACE@ZIF-8 | 10.80 | - |
| DOX@ZIF-8 | - | 2.36 |
| (DOX+ACE)@ZIF-8 | - | 0.66 |
